# Sulfur Diffusion Studies Imitating Recycled Ground-Rubber-Containing Compounds

**DOI:** 10.3390/polym16223112

**Published:** 2024-11-06

**Authors:** Stefan Frosch, Volker Herrmann, Tim Schülein, Fabian Grunert, Anke Blume

**Affiliations:** 1 Faculty of Plastics Engineering and Surveying, Technical University of Applied Sciences Würzburg-Schweinfurt, 97070 Würzburg, Germany; stefan.frosch@thws.de (S.F.); volker.herrmann@thws.de (V.H.); tim.schuelein@thws.de (T.S.); 2 Elastomer Technology and Engineering (ETE), University of Twente, 7522 NB Enschede, The Netherlands; f.grunert@utwente.nl

**Keywords:** recycling, ground rubber, sulfur diffusion, x-ray fluorescence, indentation, viscoelasticity, crosslink density

## Abstract

In-rubber properties of vulcanizates deteriorate in the presence of incorporated recycled ground rubber (GR). This behavior is partly explained by a possible diffusion of sulfur from the rubber matrix into the GR. Therefore, the sulfur concentration and, thus, the crosslink density in the matrix are reduced. This phenomenon was further investigated in this research work using two spatially resolved methods that supplement each other: the diffusion of soluble sulfur in GR-containing compounds was locally investigated via Micro X-Ray Fluorescence analysis. Viscoelastic properties were also determined spatially by the Micro Dynamic-Mechanical Indentation method. Combining the results of both methods, local concentrations of sulfur were related to local viscoelastic properties, revealing great differences in crosslink density at the interface between the GR and matrix material. In this way, it is shown that sulfur is capable of diffusing several mm, which locally doubles its concentration with respect to the sulfur content of the compound formulation. This, in turn, negatively impacts the homogeneity of crosslink density in both the matrix and GR, revealing a local increase in the elastic stiffness of 100 %. In addition, it was found that the vulcanization characteristics of the used polymers determine the amount of sulfur diffusion and, thus, the change in viscoelastic properties.

## 1. Introduction

More than 3 million tons of end-of-life tires were accumulated in Europe in 2019 [1]. To make this material available again in the sense of a circular economy, recycling is important. The incorporation of ground rubber (GR) into green compounds is one form of rubber recycling. GR is shredded vulcanized rubber from end-of-life materials with typical diameters ranging from 0.2 to 2 mm [2,3,4,5]. Unfortunately, in-rubber properties of GR-containing vulcanizates are typically deteriorated in contrast to formulations without GR. This phenomenon can partly be explained by the diffusion of free unvulcanized sulfur from the surrounding matrix material into the prevulcanized GR particles during mixing, storage, and vulcanization processes. Thus, the concentration of sulfur is increased in the GR particles and decreased in the matrix. This results in over-crosslinked GR particles and under-crosslinked matrix material [6,7,8,9,10].

Generally, there is a potential for numerous compound constituents to diffuse within one or between two different rubber specimens. For example, a number of studies have investigated the diffusion of antioxidants [11,12,13], waxes [14,15], and curatives [16,17]. Ignatz-Hoover et al. describe diffusion as ”movement of a soluble compound additive prompted by a disruption in equilibrium. […] When the concentration of a totally soluble component at the interface […] is reduced, the soluble component diffuses to re-establish concentration equilibrium” [17].

The diffusion coefficient D in mm^2^∙s^−1^ describes the mobility of a soluble component through a rubber and can be calculated [18,19,20]. Influencing factors of diffusion are temperature [21,22], distance from the interface [21], storage time [21], concentration of carbon black [23], concentration of sulfur [24], crosslink density [24], polymer and T_g_ (respectively molecular weight and mobility) [21], and the size and molecular weight of the soluble component [24].

The diffusion of sulfur from the matrix into the GR was observed, even when both phases originally contained the identical concentration of the crosslinking agent [7]. In this case, the reason for the diffusion is that the sulfur in the matrix is free and mobile, whereas the sulfur in the GR is mainly covalently bonded to the polymer chains. There is, thus, a concentration difference between mobile sulfur and bound sulfur when GR is incorporated into the green matrix. Due to covalent bonds, the bound sulfur is not mobile and, thus, does not diffuse. In contrast, the free sulfur might diffuse into the GR until the concentration equilibrium is re-established.

The sulfur added to the green compounds is intended to chemically crosslink the polymer. This results in rubber materials that are characterized by high reversible elongations. The crosslink density (CLD) indicates the “number of crosslinks per unit volume in a polymer network” [25]. In addition to other influencing factors such as curing time and curing system, it is predominantly influenced by the amount of sulfur added. Higher quantities of sulfur, therefore, form a higher CLD during vulcanization [26,27,28,29].

The CLD, in turn, determines the in-rubber properties of the vulcanizate [26]. In addition to some quasi-static properties such as tensile strength, elongation at break, and hardness, the viscoelastic properties in particular depend on the CLD. In the case of GR in a green matrix, it is likely that the inhomogeneities of sulfur concentration lead to large differences in the CLD of GR particles and the matrix when the compound is cured. This, in turn, leads to a reduction of in-rubber properties of the vulcanizate and, thus, to premature failure of the entire rubber sample.

To further investigate this effect, specimens were produced imitating the situation of GR in a green matrix. These materials were then investigated by two different analytical methods: the Micro X-Ray Fluorescence analysis (µ-XRF) and the Micro Dynamic-Mechanical Indentation method (µ-DMI). The first one is capable of measuring the sulfur signal of the specimens in a spatially resolved manner [30,31]. The second is able to detect viscoelastic properties of the specimen—also in a local manner [32]. Both methods exhibit the same resolutions between 25 and 100 µm and, thus, supplement each other.

The aim of this study was to determine the extent of soluble sulfur diffusion and to draw conclusions about the resulting dynamic-mechanical property changes. This is particularly important to better understand the in-rubber property deterioration of GR-containing compounds and to find solutions to minimize these effects. This could help to increase the recycling proportion of end-of-life rubber products. In addition, the findings may also be of interest to other areas as sulfur diffusion can also occur in, e.g., storage of rubber compounds, stacking of different plies in tire production, etc.

## 2. Materials and Methods

Parts of this chapter are literally taken from [31].

### 2.1. Compound Formulations and Mixing Conditions

Two different compounds were produced for the experiments. The samples differ in their chosen type of rubber: Natural Rubber (NR) and Styrene-Butadiene Rubber (SBR). All other components are identical, as can be found in Table 1. Soluble sulfur was chosen as a curing agent for two reasons: firstly, it has a higher capability of diffusion as insoluble (polymeric) sulfur due to its higher mobility in unvulcanized rubber [33]. This can be explained by the reduced solubility compared to insoluble sulfur [16]. Secondly, its high purity (sulfur content ≥ 99% [34]) enables improved detection via µ-XRF.

The following mixing conditions are based on the work of [2,31]: to produce the NR-samples, 1 kg of this material was masticated in an internal mixer (Werner & Pfleiderer, 1.5 l, PES3) for 20 min at 20 rpm and 25 °C.

The base compounds were produced in the same internal mixer at 40 rpm, 50 °C, and an effective volume V_eff_ of 70% in the following order:

- 0 min: Polymer,
- 1 min: ZnO, St. Acid, Carbon Black,
- 2 min 30 s: Cleaning step,
- 4 min 30 s: Dump.

The compounds were then cooled down on the laboratory mill (Schwabenthan 200 × 450; both rollers 20 rpm, gap: 2.5 mm, 40 °C) for 1 min.

After this step, the final compounds (identical conditions with base compounds) were mixed in the following order:

- 0 min: Base compound, accelerator, sulfur;
- 3 min: Dump.

After dumping and weighing the compound, it was cooled down again on the laboratory mill (front roller 16 rpm, back roller 20 rpm, gap: 2 mm at 40 °C):

0 min: Final compound on laboratory mill;

- 1 min: Cutting three times left and right, rolling up at a gap of 1 mm;
- 4 min: Change gap to 2.5 mm, set both rollers at 20 rpm; dump.

### 2.2. Rubber Process Analyzer

A SIS V50 from TA Instruments (Eschborn, Germany, former Scarabaeus) was used to determine the vulcanization curves of both samples at 155 °C. Each compound was tested three times. A heating press (Polystat 200 T from Servitec, Wustermark, Germany) was used to vulcanize 6 mm thick specimens to t_95_ + 2 min.

### 2.3. Settings µ-XRF

An M4 Tornado from Bruker Nano Analytics was used to examine the vulcanizates, which were placed on a three-dimensional movable sample table. The tube of the µ-XRF was equipped with rhodium as the target material and with no filter. The tube was set to 50 kV and 100 µA. During analysis, the sample chamber was evacuated to an absolute pressure of 20 mbar to remove the argon present in the ambient air and, thus, to increase the sulfur signal, since the fluorescence energies of both elements are very similar. The resolution (distance between two measurement spots) was set to 25 µm and the diameter of the X-ray beam to 25 µm. Every spot was exposed to radiation for 10 × 100 ms = 1 s. The corresponding fluorescence radiation was detected by two silicon-drift-detectors. The software Esprit M4 (Version: 1.6.0.286) was used for all measurements and postprocessing.

### 2.4. Settings µ-DMI

The µ-DMI is an analytical tool to determine the viscoelastic properties of rubber in a spatially resolved manner. For the study, a device was chosen that was developed in recent years at the Technical University of Applied Sciences Würzburg-Schweinfurt [32,35].

The parameters for the measurements were set as follows: a needle with a tip radius of 1 µm penetrates the rubber sample by 100 µm. During the quasi-static penetration process, the resulting force is measured. Using the detected force penetration curve, the quasi-static stiffness S_0_ can be determined. Subsequently, a sinusoidal signal (4 Hz at an amplitude of ±10 µm) is applied to the needle. Similar to a conventional Dynamic Mechanical Analysis (DMA), the viscoelastic properties are, thus, determined. The elastic component is described by the storage stiffness S′ and the viscous component by the loss stiffness S″. Analogous to DMA, the loss factor tan δ is then calculated by Equation (1). Following the sinusoidal oscillation, the needle moves out of the specimen, and the next measuring point is triggered. Here, 100 µm was selected as the resolution (distance between two measuring points) for all subsequent investigations. This enables a viscoelastic mapping of the specimen.

## 3. Results

### 3.1. Vulcanization Behavior

**Figure 1:**
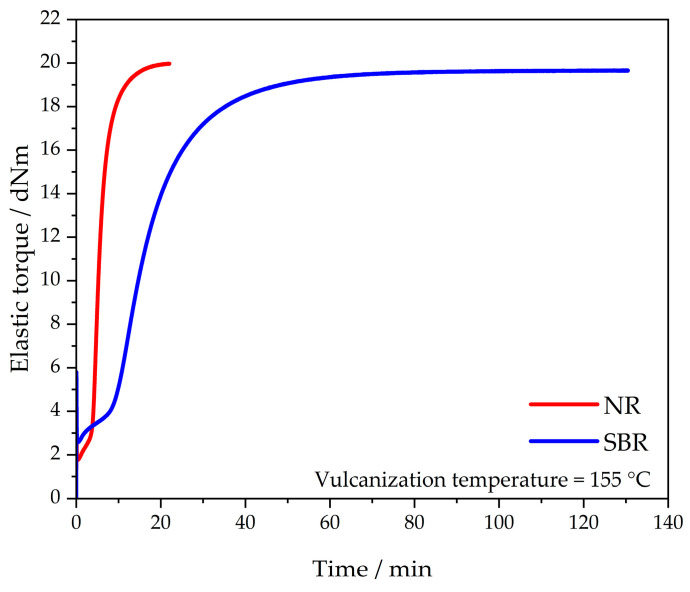
Vulcanization behavior of the NR and SBR materials.

The vulcanization behavior of the NR and SBR materials that was used for these investigations was determined three times for each. The curve of each sample material is shown in Figure 1. This is the measurement plot whose torque difference S′max–S′min represents the median of the respective three measurement curves. The measurements were stopped as soon as reversion occurred. The corresponding vulcanization times t_95_ were 12.1 min for NR and 45.0 min for SBR. The results are consistent with those determined in the literature [7].

### 3.2. Sulfur Signal Determined by µ-XRF

**Figure 2:**
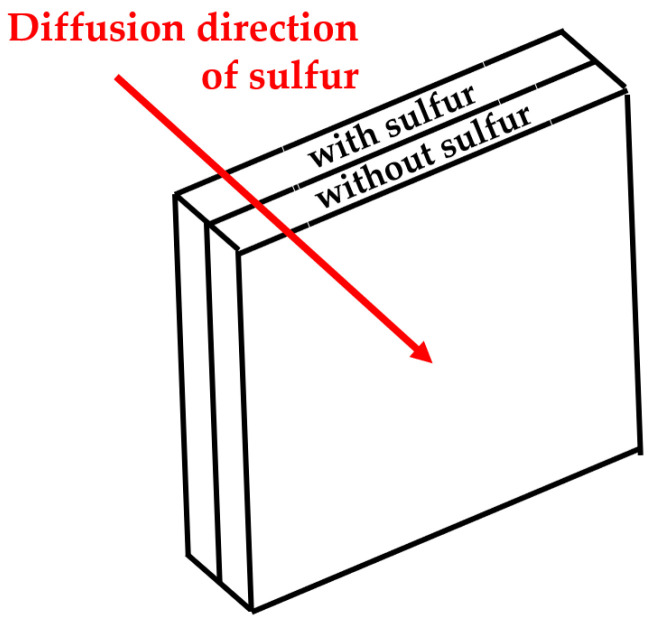
Typical diffusion setup.

The µ-XRF was used to investigate the sulfur intensity of rubber specimens and, thus, to calculate the diffusion coefficient D. A typical measurement setup for the calculation of D can be seen in Figure 2: two plates are in contact with each other. For example, one contains sulfur, and the other does not. Because the sulfur equilibrium is disturbed at the interface, the crosslinking agent diffuses from the rear plate into the front plate (see red line).

The sample preparation for the following investigations via µ-XRF and µ-DMI was based on the actual manufacturing process of the recycled samples, which is described in detail in [7,9]. To simplify, it can be stated that 10–30 parts per hundred rubber (phr) GR is added to green compounds during mixing in the internal mixer. The subsequent vulcanization step is based on the cure characteristics of the green matrix that surrounds the prevulcanized GR particles. The latter, thus, are subjected to a second crosslinking process.

**Figure 3:**
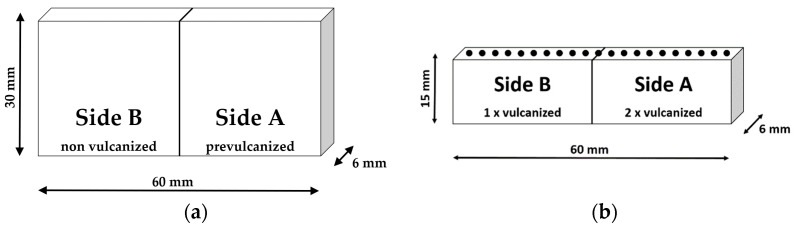
Principal structure of a composite sample; (**a**): attached sides A and B during storage procedure; (**b**): cut composite after vulcanization process (the black dots schematically indicate the measurement spots of µ-XRF and µ-DMI).

In Figure 3, a sample setup is introduced to imitate the three production steps: mixing, storage, and vulcanization of GR-containing vulcanizates. Figure 3a shows that the specimen consists of two different sides. The right side A is made of rubber already vulcanized at 155 °C to t_95_. It represents a GR particle. Side B consists of an initially unvulcanized compound, which simulates the matrix that surrounds the GR. This unvulcanized compound was initially preformed for 5 min at 40 °C and 200 bar in the heating press (Polystat 200 T from Servitec) to the dimension of 30 mm × 30 mm × 6 mm. Both sample halves A and B were placed together on one side to allow diffusion of the compound components from one side to the other. The storage was performed in the heating press at 200 bar at temperatures of 45 and 80 °C for 12 h. The different temperatures were chosen to investigate the influence of the storage temperature on the diffusion in more detail. After storage was complete, the samples were removed from the heating press and cooled to room temperature (RT).

Subsequently, the entire sample configuration was cured in the preheated heating press at 155 °C to t_95_ + 2 min of the matrix material (side B). This resulted in side A being heated a second time at the vulcanization temperature. During the curing process of the sample, it was again possible that individual compound components diffused from one side to the other.

The vulcanized specimens were then cut at half their height to prepare them for examination via µ-XRF and µ-DMI; see Figure 3b. Both analytical methods were applied in form of line scans at these freshly cut surfaces, indicated by the black dots. The measurements were performed at the cut surface representing the sample bulk and not at the actual surface. This was intended to counteract possible influences of the vulcanization skin, as this skin could have other viscoelastic properties than the bulk material. For every specimen, three line scans were conducted. Eight different specimens were produced in this manner; see Table 2.

**Figure 4:**
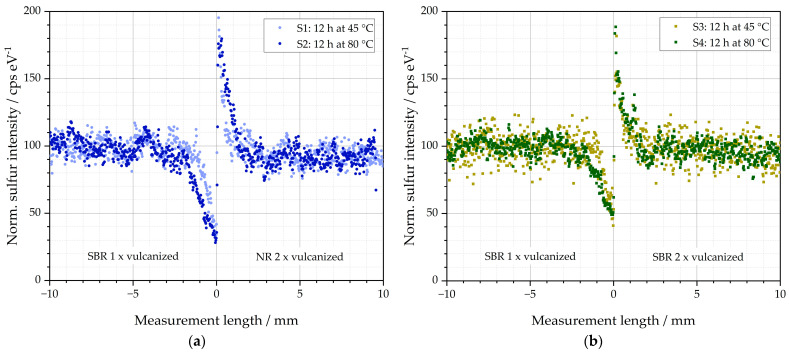
Sulfur diffusion behavior of the investigated specimens: (**a**) S1 and S2 stored for 12 h at 45 and 80 °C before subsequent vulcanization at 155 °C and (**b**) S3 and S4 stored for 12 h at 45 and 80 °C before subsequent vulcanization at 155 °C.

The µ-XRF was used to examine the samples mentioned in Table 2. The results of the elemental distributions of sulfur (S) of S1–S4 are depicted in Figure 4. The measurement length in mm is plotted on the abscissae. The value 0 is located at the exact interface of the two rubber materials of the respective specimen. Values < 0 mm describe specimen side B (matrix) and values > 0 mm specimen side A (GR). Although the samples have a total length of 60 mm (see Figure 3), only the ranges from −10 mm to +10 mm are shown in the diagrams, i.e., a total range of 20 mm. This range has been proven to be essential in pre-studies for the evaluation of the diffusion behavior under the given settings for temperature and time. The ordinate represents the normalized sulfur intensity in cps eV^−1^ (counts per second/electron Volt). Normalized in this context implies that the mean values of the sulfur intensities between the lengths of −10 mm and −5 mm were calculated to 100 cps eV^−1^ after the measurement. This simplifies the interpretation of the results since deviations from this basic intensity due to diffusion processes, for example, can be evaluated comparatively easily. In addition, the intensity changes of different specimens can be compared with each other. The normalization has no influence on the results of the following calculations, which was tested in pre-studies.

Both S1 and S2 consist of a left matrix side of SBR (one time cured) and a right side of NR-GR (cured twice). The sulfur intensities of S1 and S2 are depicted in Figure 4a. It can be concluded that the identical average sulfur intensity could be detected in both sample sides. These areas of constant average sulfur signal are between −10 and −3 mm for the SBR and between 3 and 10 mm for the NR side. This indicates that sulfur concentrations are identical in both compounds used for the left matrix and the right GR side and therefore diffusion processes due to sulfur concentration differences between both halves can be excluded.

In the interface region between −3 mm and 3 mm for sample S1, the sulfur signal is no longer constant. It decreases in the SBR from −3 mm to a minimum at the interface at 0 mm measurement length. To the right of the interface, the sulfur signal reaches a maximum in the NR. Towards greater measurement lengths between 3 and 10 mm, the sulfur signal equalizes to the base intensity at 100 cps eV^−1^. The minimum in the SBR has a value 34 cps eV^−1^ for S1. It means that the sample contains at that point only about one third of the sulfur content originally added to the sample. To the right of the interface, the maximum is 196 cps eV^−1^ and, thus, twice the sulfur concentration of the original value. Therefore, a diffusion of sulfur from left to right becomes visible: from the initially unvulcanized matrix material SBR into the initially prevulcanized NR. To the left of the interface, the rubber compound loses about 70 % of the originally added sulfur.

According to this effect, a corresponding intensity of about 170 cps eV^−1^ would have been expected locally to the right of the interface. However, the measured maximum intensity was, instead, 196 cps eV^−1^. A possible explanation for the reduced diffusion length of the right side in comparison to that of the left side could be the faster crosslinking behavior of the used NR of the right side. It converts the diffused free sulfur faster to covalent bonds between two polymer chains than the SBR. As a consequence, to the right NR-GR side, diffused sulfur is chemically bonded before it can diffuse deeper into the specimen. Therefore, the sulfur concentration increases at the close vicinity to the right of the interface.

Sample S2 consists of the same material pairing as S1 (left SBR matrix and right NR-GR). The difference is that S2 was stored at 80 °C for 12 h before crosslinking. Sample S1 was only stored at 45 °C. S2 shows similar diffusion peaks as reported for S1. However, due to the higher storage temperature, more diffusion occurs. This can be seen from the fact that the area of both peaks as well as the diffusion lengths increase.

The same behavior of sulfur diffusion can be seen for S3 and S4 in Figure 4b. In contrast to S1 and S2, the right half consists of initially prevulcanized SBR, and the left matrix side consists of initially unvulcanized SBR. The basic behavior of the sulfur diffusion appears to be identical. However, the diffusion lengths are, by about 2 mm, identical in both directions from the interface when identical materials are used in both specimen halves of S3 and S4.

**Figure 5:**
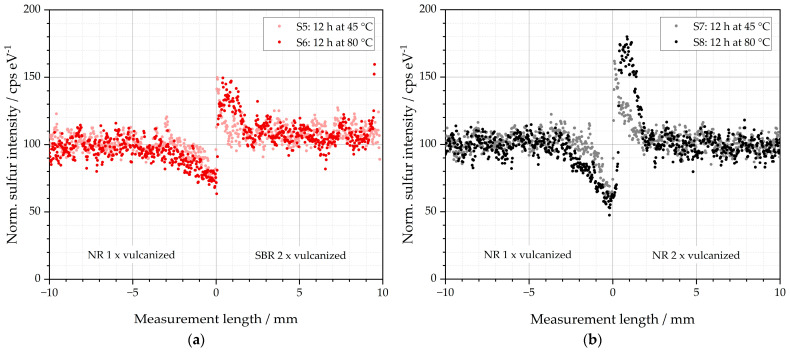
Sulfur diffusion behavior of the investigated specimens: (**a**) S5 and S6 stored for 12 h at 45 and 80 °C before subsequent vulcanization at 155 °C, and (**b**) S7 and S8 stored for 12 h at 45 and 80 °C before subsequent vulcanization at 155 °C.

The sulfur intensities of samples S5–S8 are depicted in Figure 5. These are specimens whose left sides are composed of an initial green NR compound that imitates the matrix material.

As observed above for samples S1–S4, sulfur diffusion occurs in S5-S8 from the respective left matrix into the right GR side. The storage temperature has a significant influence on the extent of this diffusion: both the intensity of the peaks and the diffusion lengths are significantly increased for S6 and S8 at storage temperatures of 80 °C compared to samples S5 and S7 at 45 °C. In addition, the material of the right GR imitating half of the specimens influences the diffusion behavior. The peak is significantly more pronounced for samples S7 and S8 with a right GR half of NR than for samples S5 and S6 with a right GR half of SBR. The diffusion behavior of the sulfur is, therefore, elevated from the left half of the NR if the right half is also NR. This is consistent with the findings above that the velocity of vulcanization also affects the amount of sulfur diffusion. It reveals that the faster vulcanization process in the NR-GR further reduces the concentration of free soluble sulfur, which, in turn, promotes the diffusion of free sulfur from the matrix into the GR to reduce the concentration difference.

In addition, it is noticeable that the peaks to the left and right of the interface differ in shape: for S5 and S7—i.e., at storage temperatures of 45 °C—the peaks in both halves display the shape known from samples S1–S4. Minima and maxima are located directly at the interface. For samples S6 and S8, however, it is noticeable that the respective peak maximum is shifted to the right half of the sample. It is approx. +1 mm for both samples. An explanation could be that at 80 °C, a more mobile sulfur species is formed on the left side of the matrix, which diffuses more easily into the prevulcanized right side. This mobile species is only formed if NR (S6 and S8) is selected as the matrix material. With SBR (S2 and S4), this species is not formed. A possible explanation could be that the proteins or other residues in the NR additionally contribute to those mobile sulfur species. Since the impurities are not in the SBR, this species cannot form in the SBR matrix.

The determined diffusion lengths of the investigated specimens are up to 2.5 mm into the already vulcanized sample half, which represents the GR material. The GR particles used in the literature differ in their size distribution, but the diameter is usually between 0.2 and 2 mm [2,3,4,5]. This clearly shows that under the present conditions, the diffusing sulfur is able to completely penetrate the recycled GR material. It, therefore, does not adhere to the surface but diffuses into the particles.

### 3.3. Comparison of the Sulfur Diffusion Behaviors by Using the Diffusion Coefficients and the Peak Area Approach

To distinguish the sulfur diffusion behavior of all samples S1-S8, the diffusion coefficient D of every specimen was determined. Their calculations are based on the explanations of [18,19,20,36]. Every specimen was measured three times with the µ-XRF. The results of the calculated sulfur diffusion coefficients are depicted in Table 3 in columns 4 and 8. The respective median value for D_(B)_ of the left sample half is highlighted in bold in column 4 and is chosen as the criterion for the displayed curves in Figure 4 and Figure 5. D_(A)_ describes the diffusion coefficient of sulfur of the right side of the sample. The errors of both Ds are specified in columns 5 and 9 in the form of the 95 % confidence interval. This means that the values of D lie within the specified error band with a probability of 95%.

Comparing the determined D_(B)_ of the left sample halves of S1, S3, S5, and S7, which were initially stored at 45° C for 12 h before vulcanization, it becomes clear that they do not differ mathematically when the errors are considered. The same applies to the right sample halves of S1, S3, and S5. Only S7 shows significantly higher D_(A)_ values.

After 12 h of storage, the samples were vulcanized at 155 °C to t_95_ of the respective left sample side. The t_95_ of NR is 12.1 min, and the t_95_ of SBR is 45.0 min. Although samples S1 and S3 (both with SBR matrix compound) were kept at 155 °C about four times longer than S5 and S7 (both with NR matrix compound), they did not exhibit a higher D and, thus, mathematically no higher sulfur diffusion. The reason for this is that the 12 h storage time is included in the calculation of D. This results in test times of 12 h + t_95_ of the respective matrix material. The diffusion during the 12 h at 45 or 80 °C, thus, outweighs the diffusion effects during crosslinking at 155 °C. It is, therefore, not possible to separate the respective effects. In addition, the 12 h storage period causes the determined D value to be about one decade lower than the comparative values from the literature [37]. The D is intended to describe the diffusion rate of a single molecule from one material into another dependent on the time. It is a result of the detected diffusion length of the solute. In contrast, the absolute quantity of the diffusing substance, which is described by the peak areas of each sample side, is less important in the mathematical approach.

**Figure 6:**
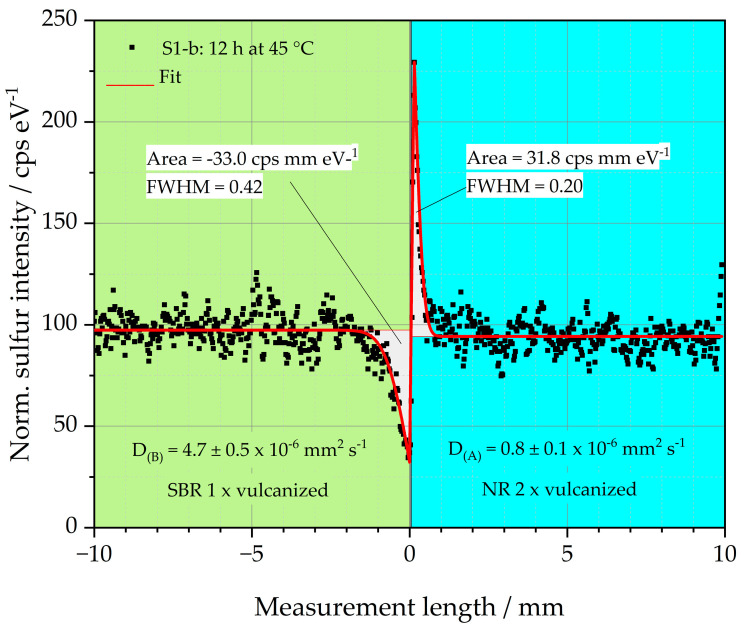
Diffusion coefficients as well as peak area approach for sample S1-b.

An example of this relationship is depicted in Figure 6. The sulfur intensity of sample S1-b is plotted over the measurement length. Sample S1-b consists of a left single crosslinked SBR matrix (green background) and a double crosslinked side of NR (blue background), which represents the GR. Before the sample was vulcanized, it was stored at 45 °C for 12 h. The black dots represent the locally measured sulfur intensity. The graph demonstrates that sulfur diffuses from the left side of the sample into the right side. The sulfur diffusion coefficients for both sample halves were determined using a fit (red line) to the black measurement points as explained in [18,19,20,36]. The sulfur diffusion coefficients D_(S)_ of both halves of the specimen are given in Figure 6. For the left sample half, D_(B)_ is 4.7 ± 0.5 × 10^−6^ mm^2^ s^−1^, and for the right sample half, the diffusion coefficient D_(A)_ is 0.8 ± 0.1 × 10^−6^ mm^2^ s^−1^. Both values are also listed in Table 3.

The results of the D_(S)_ indicate that the diffusion rate of the sulfur in the left half of the sample made of SBR is higher than in the right half of the sample made of NR. The sulfur diffusion length is, therefore, pronounced in the left half of the sample compared to the right half. The reason for the reduced diffusion length of the right sample half could be the fast vulcanization behavior of the NR, as discussed above. The diffusing sulfur is, thus, formed into covalent bonds very quickly. This happens so fast that the sulfur has no time to diffuse deeper into the right side of the sample.

Considering the peak areas of the sulfur in both halves of the sample, it becomes clear that these are similar with values between 31.8 and −33.0 cps mm eV^−1^ (negative sign due to the downward peak). The amount of sulfur mobilized to the left and right side of the interface is, therefore, constant. Due to the faster crosslinking of the right specimen half, a higher and narrower peak is formed. The Full Width at Half Maximum (FWHM) of this peak is 0.2, which is only about half the FWHM of the left half of the sample at 0.42. It is, therefore, clear that the observation of the peak geometry can support the interpretation of the results of D_(S)_.

This applies in particular if the same D_(S)_ values are determined for two different samples, as shown for S3-a and S5-b as an example. They are listed in Table 3. Both samples consist of a prevulcanized SBR half on the right. The left half of sample S3-a is made of SBR, and the left half of sample S5-b is made of NR. Both samples were stored at 45 °C for 12 h prior to vulcanization. Considering the D_(S)_ of the left sample halves (side B) of S3-a and S5-b, it becomes clear that these are the same at 6.6 ± 1.2 × 10^−6^ mm^2^s^−1^ and 6.9 ± 2.0 × 10^−6^ mm^2^s^−1^, respectively, taking the measurement error into account. Accordingly, the diffusion length of the sulfur is the same for both matrix polymers. However, when comparing the peak areas of the sulfur in both matrix systems, it becomes clear that there are significant differences in the sulfur diffusion. According to the 6^th^ column of Table 3, the peak area for sample S3-a is 28.8 cps mm eV^−1^, and for sample S5-b it is only 13.1 cps mm eV^−1^. It is, therefore, approximately twice as high in the SBR matrix (S3-a) than in a matrix made of NR (S5-b), if both systems have a right adjacent GR side made of SBR. This clearly shows that about twice as much sulfur diffuses out of the SBR matrix into an SBR-GR than out of a matrix made of NR into an SBR-GR. The fact that the sulfur loss in a matrix made of NR is lower than in one made of SBR could be explained by the faster crosslinking behavior of NR. This causes the mobile free sulfur to be bound faster, which reduces the diffusion time compared to a matrix of SBR. As a result, more sulfur diffuses out of the SBR than from an NR.

The same relationship between vulcanization properties and diffusion is valid for the right sample halves. The choice of material and, thus, vulcanization characteristics essentially determines the diffusion behavior of sulfur in the investigated specimens. Diffusion into the two-times crosslinked right GR representing half of the sample is particularly pronounced when NR is used (S1, S2, S7, and S8). If SBR (S3, S4, S5, and S6) is selected as the polymer for the right side, the peak areas are reduced, and there is less diffusion. As the vulcanization of NR-GR is about four times faster than that of SBR, the covalent bonding of the sulfur occurs faster. As a result, the concentration of free sulfur due to covalent bonding decreases in the GR faster when it is made of NR. This promotes the diffusion of more free sulfur from the matrix into the GR to counteract its concentration difference.

### 3.4. Viscoelastic Properties Determined by µ-DMI

The viscoelastic properties of S1, S2, S5, and S6 were determined by using µ-DMI. Line scans were performed on the same cut surfaces that had previously been investigated with µ-XRF; see Figure 3. However, the position of the measurement spots of the µ-DMI on this cut surface may slightly differ from those of µ-XRF, since the sample had to be transferred between both devices. Three measurements were carried out with the µ-DMI for each specimen. For better overview, only the graph is depicted in each of the following diagrams with the median value for S′ of the left sample side.

**Figure 7:**
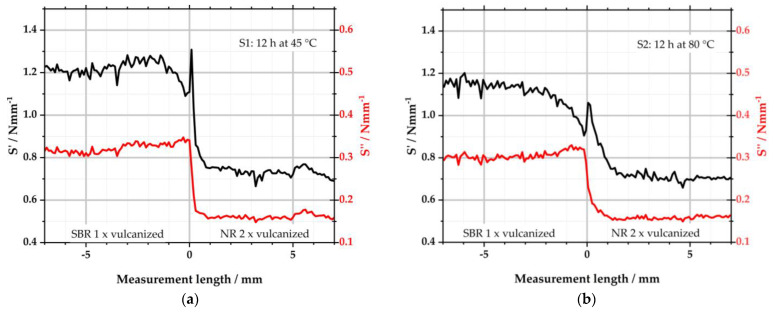
Viscoelastic properties determined with the µ-DMI: (**a**) depicts the results of sample S1; (**b**) depicts the results of S2.

Figure 7 depicts the results of S1 and S2. Both samples consist of a left, one-time-vulcanized SBR matrix side and a right two-times-vulcanized NR-GR side. They differ in the storage temperature, as S1 was stored for 12 h at 45 °C and S2 for 12 h at 80 °C prior to the subsequent vulcanization process. Like the results from µ-XRF, the measurement length is again plotted on the abscissae. Negative measurement lengths describe the left matrix side of the specimens and positive measurement lengths the right side. S′ (storage stiffness in Nmm^−1^ = elastic component) and S″ (loss stiffness in Nmm^−1^ = viscous component) are plotted on the ordinates.

From Figure 7a, it becomes clear that the viscoelastic properties of the selected polymers differ. Both S′ and S″ show higher values for the left SBR compound than for the right NR compound. This was reported before in [38]. Beyond the interface—i.e., for values between −7–−2 mm and 2–7 mm—these values are almost constant. In the interface region, however, significant differences occur: S′ starts to decrease from −1.5 mm, leading to a minimum in the left SBR matrix just before the interface. Directly right of the interface, the elastic component reaches a maximum in the NR-GR. From there, S′ reduces again over the next millimeters to level off to the constant value of NR. This behavior corresponds to the local sulfur intensity detected by µ-XRF. Due to the diffusion of sulfur from the left matrix to the right GR half of the sample, there is a local minimum to the left of the interface, while the sulfur concentration is locally increased to the right of the interface; see Figure 4a. The diffusion of the sulfur is stopped by its conversion into covalent bonds to the polymer. The detected sulfur is, therefore, no longer free but is bound at every measured point. The diffusion of the sulfur, thus, leads to local differences in the CLD. Because of the sulfur minimum on the left of the interface, less crosslinks were formed here in comparison to the constant areas (−7 to −2 mm) outside the interface. As a result, the minimum S′ was measured at this point with the µ-DMI. Since the sulfur concentration is higher to the right of the interface, the CLD is locally increased there. This leads to the maximum of the measured S′ in the NR side.

The graph of S″ partially behaves similarly to that of S′: in areas outside the interface, the viscous part adopts largely constant values. Starting from −1.5 mm, S″ takes on a maximum towards the interface, although the increase is only slightly pronounced. This is again due to the sulfur diffusion and the resulting local change in CLD. Because the sulfur concentration is reduced to the left of the interface, fewer crosslinks can form there. As a result, S′ is reduced as described above. At the same time, the viscous component S″ increases when the CLD is locally lower. On the right side, S″ almost abruptly equals the value of the right side. The trend of S″ follows that of S′. In the right NR half, the change in CLD affects both parts of the viscoelasticity equally.

Sample S2 shows a similar picture to S1, but the effects are more pronounced due to the higher storage temperature of 80 °C and the consequently pronounced sulfur diffusion as depicted in Figure 4a. S′ already starts to decrease from −2.5 mm to reach the minimum of the left side at the interface. To the right of the interface, S′ rises again to a peak and then converges within the next 2 mm to the characteristic values of the right side. The loss stiffness S″ is also constant to the left and right of the interface region. To the left of the interface, it increases steadily from −2 to 0 mm. To the right of the interface, S″ decreases to the value of NR within 1.5 mm.

**Figure 8:**
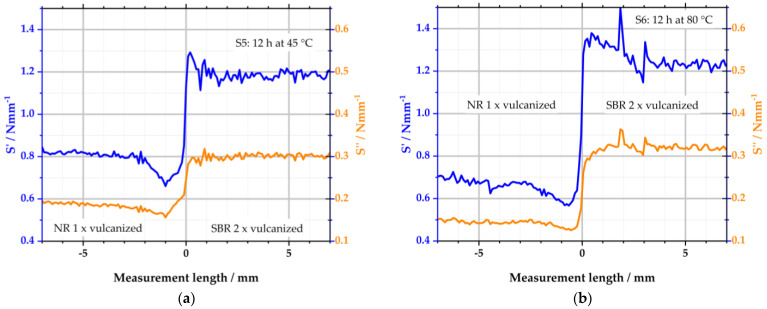
Viscoelastic properties determined with the µ-DMI: (**a**) depicts the results of sample S5; (**b**) depicts the results of S6.

Figure 8 depicts the results of samples S5 and S6. Compared to samples S1 and S2 from Figure 7, the combination of the polymers is changed: the left side of the specimens consists of one-time-vulcanized NR and the right side of two-times-vulcanized SBR.

Sample S5 displays constant values for S′ in the range of −7–−2 mm. From −2 mm, S′ decreases to a minimum at −1 mm. From there, S’ increases again. To the right of the interface, S′ reaches a maximum. Within the next 2 mm, S’ equals the level of the right side of the specimen. The changes in S′ over the measuring length can again be explained by the variation in the local sulfur concentration and the resulting local change in CLD. S′ is lower to the left of the interface because the sulfur concentration is significantly reduced there. To the right of the interface, S′ increases accordingly because the local maximum of the sulfur content is detected there. S″ is also constant outside the interface. At −1 mm, the viscous component reaches a local minimum. No changes in S″ are detectable to the right of the interface. The constant level of S″ in the right half of the sample is because the rubber was already vulcanized to t_95_ before the 12 h storage period. Accordingly, S″ was already predetermined. The increase in sulfur in the right SBR half, therefore, only has an influence on the storage stiffness S′.

S6 shows a similar behavior as S5; see Figure 8b. However, the increased storage temperature of 80 °C promotes the sulfur diffusion and, thus, the alterations of viscoelastic properties. It becomes clear that the reduction of S′ starts at −3 mm. This indicates that the diffusion length increased in comparison to the 2 mm of S5, which was stored at 45 °C. To the right of the interface, S′ does not reach a constant value before 3 mm of measurement length, which is also increased to the value of 2 mm of S5. The peak is more pronounced than the peak of S5. S6 has a singular peak at 2 mm with about 1.5 Nmm^−1^. The peak is the result of a single measurement spot and is, therefore, presumably a measurement artifact or an error in the evaluation of this spot in the measurement software. The behavior of S″ is comparable to that of sample S5, including the described temperature-promoting effect.

### 3.5. Correlation of Sulfur Diffusion and Viscoelastic Property Changes

**Figure 9:**
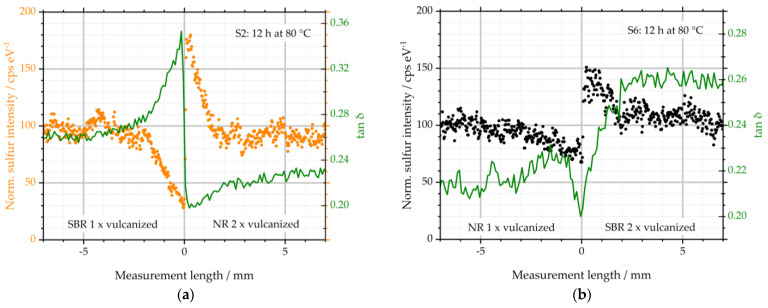
Comparison of sulfur diffusion and loss factor tan δ: (**a**) depicts the results of sample S2; (**b**) depicts the results of S6.

It was found that for the investigated specimens, sulfur diffusion occurred in the interface region. At the same time, differences in viscoelastic properties could be detected. In the following discussion, the results of both analytical methods µ-XRF and µ-DMI are combined. This is depicted in Figure 9 for specimens S2 and S6.

The respective measurement lengths are plotted on the abscissae. The normalized sulfur intensity can be seen on the left ordinate. The points are taken from Figure 4 and Figure 5. On the right ordinate, the loss factor tan δ is plotted. It is chosen to be displayed, as it combines S′ and S″ according to equation 1 in a single value and is, thus, suitable for a concise comparison of the results from µ-XRF and µ-DMI.

tan δ = (S″) ∙ (S′)^−1^ (1)

For sample S2, it becomes evident that the sulfur intensity correlates with tan δ in a reciprocal manner. Between −2.5 and 0 mm, the sulfur concentration decreases significantly. This behavior is due to the diffusion of the crosslinking agent from the left into the right half of the specimen. According to this, tan δ on the left of the interface increases with decreasing sulfur intensity and, therefore, reduced CLD. The maximum of tan δ is, thus, at the interface of the specimen.

To the right of the interface of S2, the behavior is opposite: due to the described diffusion, the sulfur intensities are increased there. This local rise leads to an increase in CLD, which can be stated by the minimum of tan δ directly to the right of the interface. The sulfur intensity and viscoelastic properties both reach constant levels as the measurement length increases. A similar behavior could be observed in analogy for S1. However, due to the lower storage temperature, the described effects are reduced to slightly shorter lengths, beginning with the increase in tan δ at −1.5 mm.

As depicted in Figure 9b, S6 shows a similar behavior to that of sample S2: the change in sulfur intensity from the left side of the sample into the right side reveals the diffusion of sulfur. These local concentration differences of the crosslinking chemical cause local differences in CLD. To the left of the interface, tan δ increases accordingly. In the double-vulcanized right half, which represents the GR, the CLD increases. As a result, tan δ shows a minimum.

The extent of the diffusion length—approx. 2.5 mm each to the left and to the right of the interface—indicates that GR with typical sizes of a maximum of 2 mm can be completely enriched by sulfur. Accordingly, these particles have lower tan δ than the surrounding matrix due to a local rise in CLD inside the GR particles and a reduction of CLD in the matrix material. As a result, the homogeneity of the GR-containing vulcanizates decreases.

The matrix determines mainly the in-rubber properties of a GR-particles-containing vulcanizate, since the volumetric proportion of the matrix is higher than that of the GR. The sulfur diffusion reduces the CLD of the matrix, which leads to a change in its in-rubber properties. This effect is supported by the fact that the CLD of the GR particles increases. This leads to a rise in the intrinsic elongation of the matrix in the immediate vicinity of the GR under load. If the local stress in this area exceeds the matrix maximum tensile strength, which has already been reduced by the diffusion of the sulfur, the matrix material fails and, thus, the entire vulcanizate is affected. It is, therefore, highly likely that the diffusion process caused by the use of GR is the main reason for the deterioration of the in-rubber properties of a GR-particles-containing vulcanizate. This is consistent with the findings of Kim et al. [39].

## 4. Discussion

The incorporation of ground rubber in green compounds leads to a deterioration of the in-rubber properties of the vulcanizate. According to the literature, this is partly explained by the diffusion of sulfur from the matrix into the particles, reducing the CLD of the matrix [6,7].

This effect was reproduced and further investigated in this work. It was shown that the concentration of sulfur in the matrix material decreased while it locally increased in the GR-imitating material, although both sides had identical concentrations of sulfur before the start of the diffusion experiment. The driving force for diffusion was the difference in the concentration of free sulfur in the two specimen halves before the start of the investigation. Because most of the sulfur is bound to the polymer in the right GR-imitating half, which is prevulcanized to t_95_, the concentration of free sulfur is low. In contrast, it is high in the green left half because no crosslinking has yet taken place. During storage at 45 or 80 °C, the difference in concentration of the free sulfur between both sides is reduced as sulfur diffuses from the left to the right side of the specimen. Furthermore, the free sulfur can also diffuse at the vulcanization temperature of 155 °C.

A higher storage temperature promotes the diffusion of sulfur, as it increases the mobility of both sulfur and the polymer. In practice, this means that significant quantities of sulfur diffuse between the matrix and the GR particles not only during storage, but especially at the elevated temperatures during compounding.

The investigations also showed that the diffusion depends not only on the temperature but also on the selected polymers of the two sides. Using the calculated diffusion parameters and peak areas, it was found that more sulfur diffuses from a left matrix side made of SBR than from one made of NR. This is due to the slower vulcanization process of SBR compared to NR. Accordingly, the free sulfur present in the SBR matrix is bound more slowly than in NR. This extends the time window in which it can diffuse. As a result, the sulfur diffusion from a left SBR side is increased compared to a side made of NR.

It was additionally revealed that the choice of GR material also determines the extent of diffusion. If the fast crosslinking NR is selected for the right GR-imitating side of the specimen, a particularly large amount of sulfur diffuses from the matrix into the GR. This is because the diffusing free sulfur is quickly bound to the polymer in the form of a crosslink. Consequently, the concentration of free sulfur in the right half of the sample decreases. This, in turn, ensures that the diffusion of the sulfur from the left to the right side is increased to compensate for the concentration gradient.

Measurements with the µ-DMI show that the viscoelastic properties of both sides of the specimen are also changed in the area of the interface. S’ of the matrix is reduced in the vicinity of the interface, while it is increased in the GR-imitating material. Therefore, changes in the elastic components could be assigned to the identical areas whose sulfur diffusion had previously been investigated using µ-XRF. It is, therefore, clear that the diffusion of the sulfur from the left matrix side into the right GR side determines the change in the viscoelastic properties. As a result, the CLD is reduced on the left side, while it is increased on the right side. If the picture is transferred to the GR-containing samples, it becomes clear that the sulfur diffusion is responsible for the reduction of the in-rubber properties of the vulcanizate.

## 5. Conclusions

The large quantities of end-of-life tires produced globally pose a major challenge in terms of sustainability. One way to recycle these tires is to turn them into ground rubber, which is then added to virgin compounds as feedstock. However, this results in sulfur diffusion from the green matrix into the vulcanized ground rubber particles, which deteriorates the properties of the entire vulcanizate. In the current study, this effect was investigated in more detail by using spatially resolved analytical methods.

It was found that due to diffusion, the sulfur concentration in the ground rubber can double locally, which causes a local increase in the elastic proportion in a considerable manner. A direct quantitative correlation between sulfur diffusion and the change in viscoelastic properties could, thus, be demonstrated. It was also shown that the vulcanization characteristics have a decisive influence on the extent of diffusion and, thus, the viscoelastic properties. A faster crosslinking process covalently bonds the sulfur in a shorter time, which reduces the sulfur mobility and, thus, the ability to diffuse from the matrix into the ground rubber.

## Figures and Tables

**Table 1:** Compound formulations; all values in parts per hundred rubber (phr).

	NR	SBR	Name	Company
NR	100	-	SIR 20	Weber & Schaer GmbH & Co. KG, Hamburg, Germany
E-SBR	-	100	Buna SE 1502 L	Arlanxeo Deutschland GmbH, Cologne, Germany
Carbon Black	50	50	Corax N330	Orion Engineered Carbons S.A., Eschborn, Germany
ZnO	5	5	Zinkoxid Rotsiegel	L. Brüg ge mann GmbH & Co. KG, Heilbronn, Germany
Stearic Acid	3	3	Edenor ST1 GS	KLK Emmerich GmbH, Emmerich am Rhein, Germany
Soluble Sulfur	1.2	1.2	K46859483 542	Merck Chemicals GmbH, Darmstadt, Germany
Accelerator	1.2	1.2	Vulkacit CZ/EG-C	Lanxess Deutschland GmbH, Krefeld, Germany

**Table 2:** Overview of the composition of the composite samples.

Specimen	Temperature/°C	Side B (1 × Vulcanized)	Side A (2 × Vulcanized)
S1	45	SBR	NR
S2	80	SBR	NR
S3	45	SBR	SBR
S4	80	SBR	SBR
S5	45	NR	SBR
S6	80	NR	SBR
S7	45	NR	NR
S8	80	NR	NR

**Table 3:** Calculated sulfur diffusion coefficients of samples S1–S8; the median value of each sample for the left side B is printed in **bold**.

		Side B (1 × Vulcanized)				Side A (2 × Vulcanized)			
Specimen	T/°C	Polymer	D_(B)_/10^−6^ mm^2^ s^−1^	95% conf./E^−6^	Peak Area /cps mm eV^−1^	Polymer	D_(A)_/10^−6^ mm^2^ s^−1^	95% conf./E^−6^	Peak Area /cps mm eV^−1^
**S1-a**	45	SBR	**6.2**	**0.7**	39.9	NR	3.2	0.3	35.5
S1-b			4.7	0.5	33.0		0.8	0.1	31.8
S1-c			7.6	1.0	36.9		2.8	0.3	38.1
S2-a	80	SBR	21.0	1.8	67.1	NR	8.9	0.6	66.4
S2-b			21.8	1.5	71.7		10.3	0.6	73.6
**S2-c**			**21.7**	**1.4**	78.1		11.1	0.6	73.9
S3-a	45	SBR	6.6	1.2	28.8	SBR	2.2	0.3	28.6
S3-b			5.2	0.9	27.9		4.2	0.6	33.8
**S3-c**			**6.6**	**1.0**	28.9		6.3	0.9	30.3
S4-a	80	SBR	13.7	1.4	40.0	SBR	12.1	1.2	39.4
S4-b			17.4	1.6	44.0		12.9	0.8	62.2
**S4-c**			**17.2**	**1.4**	54.0		8.9	0.6	53.4
**S5-a**	45	NR	**5.1**	**1.1**	12.4	SBR	0.8	0.1	13.3
S5-b			6.9	2.0	13.1		7.3	2.1	12.8
S5-c			3.0	0.6	15.0		1.6	0.3	14.6
S6-a	80	NR	71.2	10.2	43.1	SBR	31.1	3.2	45.1
**S6-b**			**58.3**	**8.6**	42.8		22.8	2.8	43.0
S6-c			51.6	7.7	44.4		22.7	2.9	44.9
S7-a	45	NR	8.1	0.8	31.5	NR	9.9	0.9	36.4
S7-b			11.0	1.0	36.2		12.4	1.1	38.1
**S7-c**			**9.1**	**1.0**	33.1		10.1	0.9	38.8
S8-a	80	NR	39.9	3.7	72.1	NR	18.0	1.1	88.5
S8-b			34.0	3.0	69.4		15.0	1.1	77.5
**S8-c**			**38.7**	**3.3**	76.3		11.1	0.8	82.0

## Data Availability

The original contributions presented in the study are included in the article; further inquiries can be directed to the corresponding author.
